# Motor synchrony, social learning and closeness in group play settings

**DOI:** 10.3389/fpsyg.2025.1595908

**Published:** 2025-09-04

**Authors:** Trinh Nguyen, Bahar Tunçgenç, Lauren Marsh, Gabriela Markova, Lisa Horn, Nadine Pointner, Hanna Schleihauf, Stefanie Hoehl

**Affiliations:** ^1^Department of Developmental and Educational Psychology, University of Vienna, Vienna, Austria; ^2^Neuroscience of Perception and Action Lab, Italian Institute of Technology, Rome, Italy; ^3^Department of Developmental Psychology and Biopsychology, Heidelberg University, Heidelberg, Germany; ^4^Department of Psychology, Nottingham Trent University, Nottingham, United Kingdom; ^5^Social Body Lab, Institute of Human Sciences, University of Oxford, Oxford, United Kingdom; ^6^School of Psychology, University of Nottingham, University Park Campus, Nottingham, United Kingdom; ^7^Institute for Early Life Care, Paracelsus Medical University, Salzburg, Austria; ^8^Department of Behavioral and Cognitive Biology, University of Vienna, Vienna, Austria; ^9^Department of Pediatrics and Adolescent Medicine, Medical University of Vienna, Vienna, Austria; ^10^Department of Developmental Psychology, Utrecht University, Utrecht, Netherlands

**Keywords:** synchrony, imitation, development, affiliation, sharing, prosociality, group, preschool

## Abstract

**Introduction:**

Playful activities provide critical opportunities for rhythmic interactions, which may affect social and cognitive development in early childhood. Prior research suggests that motor synchrony promotes closeness and prosocial behaviour, but few studies have examined its role in social learning and in group settings.

**Method:**

This study investigated whether motor synchrony in a clapping game, enhances preschoolers' closeness with others, imitation, over-imitation, and sharing behaviour. In a group setting, motor synchrony and asynchrony were experimentally induced between the child and two experimenters. We hypothesized that children would feel closer, imitate more, and share more with an adult partner who moved in synchrony compared to one who moved asynchronously.

**Results:**

Bayesian analyses revealed no credible evidence that the children affiliated, imitated, over-imitated, or shared differently with their synchronous vs. asynchronous partner (BF10 = 0.045–0.216). Manipulation checks indicated that although the adults adhered to the stimuli, there was overall low motor synchrony.

**Discussion:**

These findings highlight the challenges of inducing motor synchrony in playful group settings and raise questions about the level of synchrony necessary to impact social affiliation and learning in young children.

## 1 Introduction

From birth, children rapidly acquire knowledge and skills through interactions with others (Westermann et al., 2007). Play and games, in their many forms, provide an essential context where children engage in social learning, imitating and learning from the actions of others (Meltzoff et al., 2009; Whiten et al., 2009; Elkind, 2008; Singer et al., 2006). A key aspect of these interactions is motor synchrony—where individuals spontaneously coordinate their movements with one another. In preschool-aged children, such synchrony is often observed during activities like dancing, clapping games, or collaborative building games (Trainor and Cirelli, 2015). Despite its suggested importance, the specific role that these synchronized activities play in social learning remains unknown.

One of the key factors that underwrite social learning lies in the high-fidelity imitation of others' actions (Heyes, 2018). When it comes to learning through copying others' actions, it has been shown that children begin to imitate an adult model's inefficient actions during their second year of life (Buttelmann et al., 2013; Nielsen, 2006). Starting at around age three (McGuigan and Whiten, 2009), children even tend to copy non-functional actions that are not strictly necessary to reach the overt goal of an action sequence [e.g., stroking the lid of a box before opening it (Marsh et al., 2014)]. This phenomenon, also known as “over-imitation,” has been associated with learning about instrumental functions of objects, learning about social norms, fostering group cohesion, and forming social bonds (Legare, 2017; Nielsen, 2018), and therefore is thought to play a crucial role in the transmission of cultural and ritualistic behavior (Henrich and Henrich, 2010).

While children are largely motivated to copy a model's behaviour, there is variation in imitation rates of functional and non-functional actions (Nielsen and Blank, 2011; Schleihauf and Hoehl, 2020). Factors concerning children's social engagement and affiliation motives influence how much a child (over-) imitates a model (Hoehl et al., 2018; Over and Carpenter, 2013). More imitation was observed following being primed with ostracism (Over and Carpenter, 2009), of in- vs. out-group members (Buttelmann et al., 2013; Kinzler et al., 2011), of communicative vs. uncommunicative models even after causal links were clear to the children (Hoehl et al., 2013). In addition, third-party expectations of affiliation are present when five-year-old children observe others imitate each other (Over and Carpenter, 2015) or 15-month-old infants observe others synchronize with each other (Fawcett and Tunçgenç, 2017). However, unexpectedly, little is known about the malleability of social motivations driving the imitation of specific interaction partners. Children's social motivations to (over-)imitate have previously been probed using group manipulations and cooperation games (see Hoehl et al., 2018, for a review). Given that (over-)imitation is more likely to occur when children have stronger social motivations (Over and Carpenter, 2013), we posit that rhythmic interpersonal coordination, in the form of motor synchrony, could be an effective promoter of social motivation and affiliation resulting in increased imitation of functional and non-functional actions.

Motor synchrony, defined as individuals matching the timing of their movements (Sebanz et al., 2005), is a commonplace feature of our everyday social interactions. For instance, audience applauses often spontaneously turn into synchronized clapping (Néda et al., 2000), but we can also deliberately coordinate our movements with others during dance (Ehrenreich, 2006). Moving together in time guides us in our social encounters and is essential in preparing and guiding social attention and motivation in early childhood (Rauchbauer and Grosbras, 2020; Rolf et al., 2009). The ability to coordinate with others and external rhythmical stimuli emerges early in childhood (McAuley et al., 2006; Provasi and Bobin-Bègue, 2003). Infants and children can actively and selectively coordinate their actions in different contexts and with different agents. Although infants start to move their bodies differently according to different tempi of musical and rhythmic stimuli at 5 months of age (Rocha et al., 2021; Zentner and Eerola, 2010), these movements are not precisely synchronized to the external stimuli. At 2.5 years of age, toddlers start to show tempo flexibility and may have moments of synchronization, especially in social contexts (Kirschner and Tomasello, 2010). However, in general, it is not until age 4 that children become more proficient in adjusting to different tempi when synchronizing with external stimuli (Provasi and Bobin-Bègue, 2003).

A growing body of research has demonstrated that experiencing motor synchrony induces increased helping and sharing in infants and young children (Rabinowitch and Knafo-Noam, 2015; Rabinowitch and Meltzoff, 2017; Tunçgenç and Cohen, 2016, 2018). In a study, 14-month-olds who were bounced synchronously with an adult showed increased instrumental helping behavior toward the bouncing partner compared to an asynchronous adult (Cirelli et al., 2014a). Furthermore, toddlers extended their help to the affiliates of the synchronized partners (Cirelli et al., 2016) but not to neutral others (Cirelli et al., 2014b). In peer interactions, synchronous play as compared to non-synchronous play also resulted in children showing more helping behavior thereafter (Rabinowitch and Knafo-Noam, 2015; Tunçgenç and Cohen, 2016). However, in some studies the joint rhythmic experience, irrespective of being synchronous or asynchronous, seemed to drive the observed prosocial effects (Rabinowitch and Meltzoff, 2017). Therefore, the question remains whether the effects of motor synchrony during play can be corroborated with other, more costly facets of prosociality, such as the distribution of resources.

Cognitive and evolutionary theories describe various mechanisms underlying the social effects of rhythmic coordination. Motor synchrony, similar to effects found in progressive behaviormatching [i.e., mimicry (Lakin and Chartrand, 2003)], supports positive affect, trust, as well as engagement through the alignment of the interactants' emotional states and representations (Sebanz et al., 2005; Baimel et al., 2015; Hove, 2008; Reddish et al., 2014). Moving in synchrony further reduces the load on socio-cognitive processing, allowing us to focus on the task at hand or on the other person (Heggli et al., 2019; Koban et al., 2019; Wheatley et al., 2012). The inherently rewarding nature of synchronous rhythmic interactions is another potentially important facet that may relate to feelings of affiliation (Hoehl et al., 2021). It has been suggested that joint actions, such as motor synchrony, increase interacting partners' sense of mutual commitment (Michael and Székely, 2018), creating the expectation that they will engage in future reciprocal exchanges. By strengthening such expectations, interpersonal motor synchrony may also promote teaching and learning (Sebanz et al., 2005; Michael and Székely, 2018; Lang et al., 2016; Mitkidis et al., 2015). Engaging in synchronous interactions, which are highly structured, may guide interacting partners to perceive the other person as reliable, and trustworthy and to provide useful information about how to attain one's desired goal.

Despite these theoretical considerations, the link between interpersonal motor synchrony and social learning has been surprisingly neglected in research on early human development. In a study with school-aged children, the degree of experienced synchrony in postural sway between two children was related to the perception of increased social competence (Vink et al., 2017). Nonetheless, it remains unclear whether children use the social information derived from interpersonal motor synchrony to inform their learning behaviour.

In the present study, we experimentally manipulated motor synchrony between preschool-aged children and two interaction partners in a playful context. While one experimenter moved in synchrony with the child, the other experimenter concurrently moved in asynchrony with the child during a previously established clapping game (Tunçgenç and Cohen, 2018; Qian et al., 2020). Subsequently, we tested whether children preferentially (over-) imitated the synchronous vs. asynchronous interaction partner and whether they showed preferential sharing toward the synchronous vs. the asynchronous interaction partner. Affiliation toward both partners was assessed before and after the motor synchrony manipulation to delineate the social affiliation effects of motor synchrony.

We aimed to tease apart the potential effects of motor synchrony on different aspects of social learning, i.e., imitation of functional manner actions and over-imitation of non-functional manner actions. The (functional) actions in this study were directed toward the same goal but differed in the mannerism of each interaction partner (Howard et al., 2015). On the one hand, motor synchrony may tap into social learning by guiding the children's attention to synchronous (as opposed to asynchronous) others, possibly making them seem more trustworthy and reliable. This effect would then facilitate the imitation of functional actions, while not necessarily affecting the imitation of non-functional actions (i.e., over-imitation). This effect should also be independent of feeling affiliated. On the other hand, if motor synchrony taps into social affiliation, we would expect motor synchrony to enhance the imitation of both functional and non-functional manner actions (over-imitation).

In addition, we predicted that if motor synchrony increases affiliation, this should guide which interaction partner (i.e., synchronous vs. asynchronous) the children prefer, resulting in them sharing more resources with the synchronous as compared to the asynchronous interaction partner. Preschool-aged children are especially suitable to study the potential link between motor synchrony and social learning, as previous research shows them to be able to synchronize precisely with others (Rabinowitch and Meltzoff, 2017; Tunçgenç and Cohen, 2018) while they also start to consistently show over-imitation at this age (Hoehl et al., 2018). By examining these dynamics, this study aims to contribute to our understanding of how play-based motor synchrony influences social learning in early childhood, with potential implications for educational practices and the promotion of social development through play.

## 2 Methods

This study was conducted as part of a registered report: https://doi.org/10.17605/OSF.IO/Z37EC.

### 2.1 Sample characteristics

We tested 75 children with an average age of 5.5 years/66.09 months (SD = 6.338 months, range = 52–80 months, 36 girls) in the current study, while utilizing sequential Bayes Factor (BF) analyses [see Section 2.4 for more details (Schönbrodt and Wagenmakers, 2018)]. We had to exclude 11 additional children because they did not adhere to the task instructions. Children were recruited from various kindergartens and a database of volunteer families and either participated at the respective kindergarten (*n* = 60) or the University lab (*n* = 15). A priori sample size range followed a power analysis reported in Section 2.5 below. The selection of this age range was decided upon after piloting 17 children aged 4 to 6.5 to ensure that the children can successfully synchronize their movements to an external rhythm and follow the movements of others simultaneously. Pilot testing in a between-subjects design (i.e., the child was either paired with a synchronous experimenter or with an asynchronous experimenter) confirmed that these beats were easy to follow and sufficiently different from each other to establish pairwise asynchrony. We piloted each task for feasibility and are not reporting statistical analyses as all tasks were adapted following the pilot study. We expected an attrition rate of around 10% according to previous studies (Tunçgenç and Cohen, 2018; Schleihauf et al., 2019). Ethical clearance was granted by the local ethics committee. Accordingly, we asked for full written informed consent from parents and verbal consent from children before taking part in the study.

### 2.2 Materials and procedure

**Figure 1:**
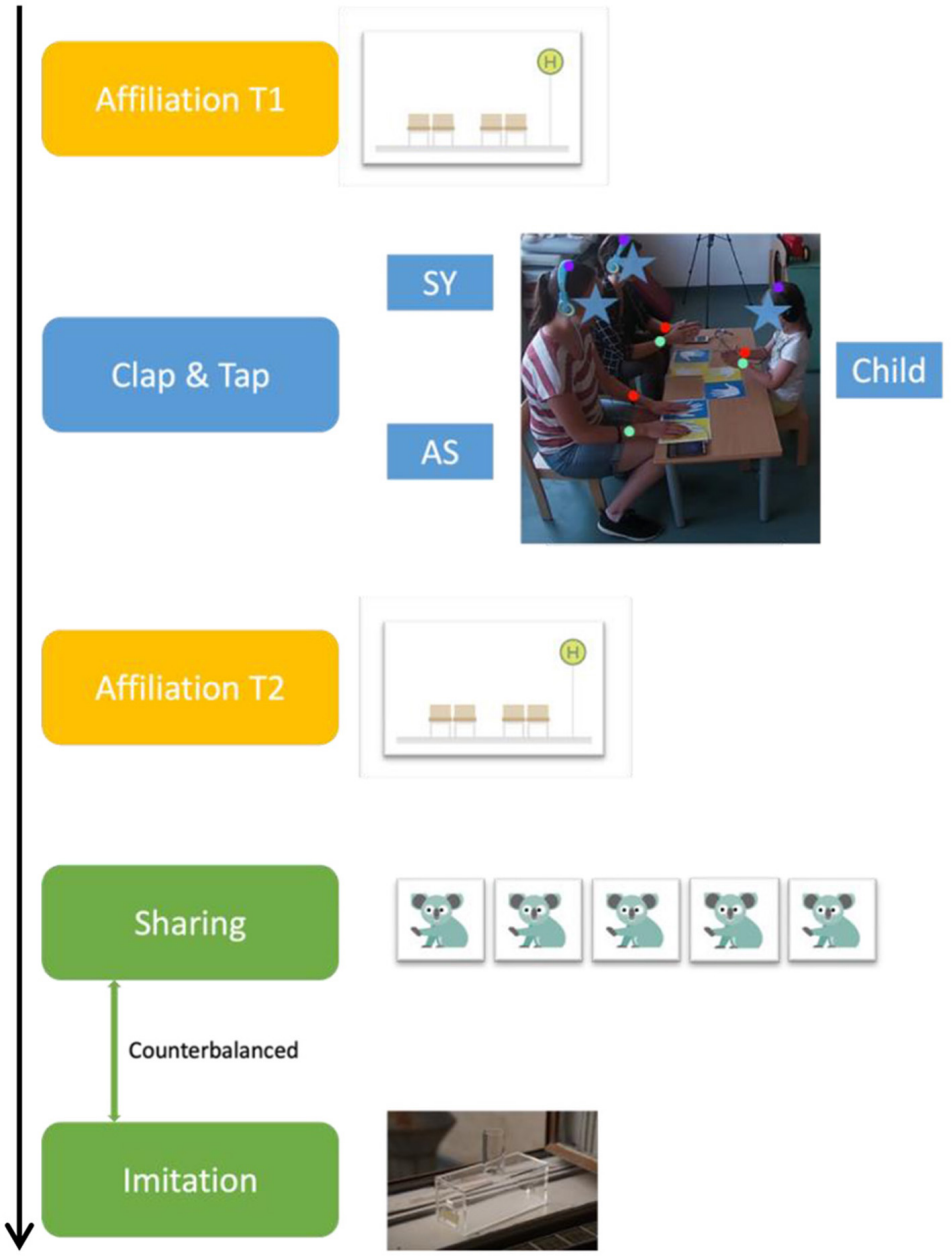
Schematic summary of the experimental procedure (top to bottom).

The experimental procedure comprised a warm-up activity (Coloring animal templates) and four main games: To measure affiliation (Bus Stop Game), imitation (Fish Box Imitation Game), and sharing (Sticker Sharing Game) or to induce motor synchrony and asynchrony (Clap & Tap Game, and; see Figure 1 for a schematic outline of the experiment). Each session took place in a quiet room with only the child, and the two experimenters present. Synchrony and asynchrony (independent variable) were simultaneously induced for two different female experimenters (age range: 22–27, White European) using the Clap & Tap Game. For each child, the assignment of the SY and AS roles to the experimenters was determined randomly to control for potential experimenter effects. Manipulation check for synchrony was assessed using video-based automated motion tracking of the children's and experimenters' hands. The following dependent variables were measured: *Affiliation* was assessed through how close the children wished to sit to the experimenters in a Bus Stop Game. Next, *social learning* was measured as the degree of imitation, namely imitation of a functional manner action and over-imitation of non-functional manner actions in a Fish Box Imitation Game. *Sharing* was assessed using a Dictator's Game (Benenson et al., 2007; Steinbeis and Over, 2017). Detailed information on the training phases and instructions for the main tasks are provided in the Supplementary material. All sessions were video recorded and subsequently coded offline.

#### 2.2.1 Affiliation measure

**Figure 2:**
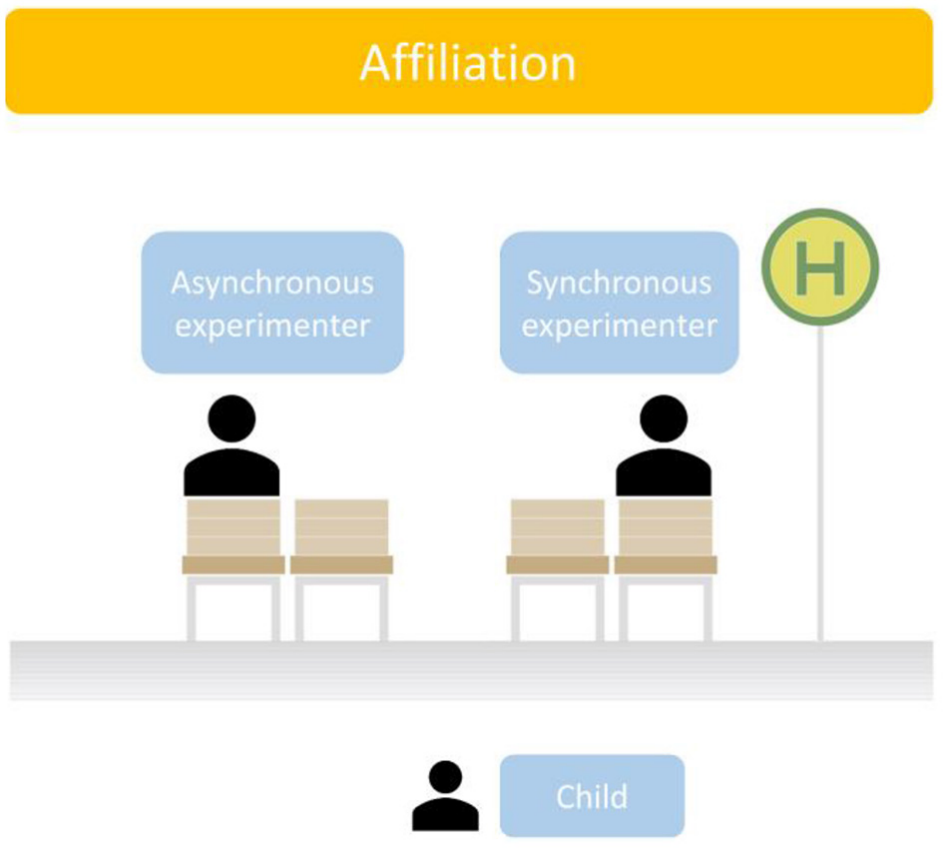
Affiliation is assessed using the Bus Stop Game. The leftmost and rightmost chairs will be occupied by the experimenters, and the child will be instructed to choose to sit down on one of the remaining chairs.

After the warm-up activity with the two experimenters, children engaged in the Bus Stop Game to indicate their initial preference for an experimenter. Children were shown a row of four chairs, with two chairs grouped and a gap between the two groups (see Figure 2). The synchronous (SY) and the asynchronous experimenters (AS) sat down on the far left chair and the far right chair. The allocation of the sides of their chairs was counterbalanced. We asked the children to choose to sit down on one of the two empty chairs. Physical Proximity to either SY or AS was taken as a proxy for social closeness. The measure was collected twice, once before (Affiliation at T1) and once after the Clap & Tap Game (Affiliation at T2).

#### 2.2.2 Motor synchrony manipulation

Following the first Affiliation Measure, the Clap & Tap Game adapted from (Tunçgenç and Cohen, 2018) commenced. Children and both the SY and AS experimenters performed specific movements in time to tick-tock sounds that they heard from their headphones for 55 s. The audio track was composed of four 10-s intervals of tick-tock sounds. At each beat of the tick-tock sounds, the child, SY, and AS alternately clapped (tick) and tapped (tock) with both hands on a “hands sheet” in front of them. Deviations from the pairing of movements and sound (i.e., clap at tock and tap at tick) were allowed as long as both child and SY moved synchronously. The four intervals were separated by a whoop sound, during which the participants performed “jazz hands” by rapidly waving both hands simultaneously before continuing with the clapping and tapping. We included the whoop sound to make the game more interesting and to add auditory emphasis to the movement activity. The choice of movements was based on previous research that has confirmed young children's synchronization proficiency in clapping and tapping (Tunçgenç and Cohen, 2018; Qian et al., 2020).

Both the child and SY heard and moved to tick-tock beats at the same tempo, while AS heard and moved to another tempo. The tempi of 100 beats per minute (BPM) and 130 BPM were counterbalanced across the SY-child dyad and AS. The three people were clapping and tapping simultaneously, with the child moving synchronously with one experimenter and asynchronously with the other throughout. The tempi were age-appropriate to the children's synchronization ability range (Provasi and Bobin-Bègue, 2003). We used a Python-based script to ensure the two different audio tracks would start at the same time and deliver those through two different sound cards. The two synchronous sound streams were delivered through an audio splitter attached to the same sound card. All three headphones were thus connected via wires to a tablet in the middle of the table. Successful motion synchronization and synchronization were assessed using video-based automated motion tracking [DeepLabCut (Mathis et al., 2018; Nath et al., 2019)] of all interactants' wrists, due to technical issues with the motion trackers originally planned for assessing motor synchrony (MbientLab, San Francisco). Motion synchrony was assessed via phase coherence of the children's and the experimenters' motion time series. We used these measures of synchrony as a group-based manipulation check to test if children moved in synchrony with one but not the other interaction partner. The results are reported in the supplements. Each child was instructed and trained by both experimenters before starting the game. Both experimenters gave an equal number of instructions and took turns giving the instructions (see Supplementary material for detailed instructions). Training began with the experimenters demonstrating how to perform the moves and continued until the child successfully performed the moves in time to the beats for half of the auditory track, i.e., 20 s. Importantly, the experimenters only showcased the moves individually, and never at the same time with each other or with the child. After training, the child sat across both experimenters to perform the activity together in the test phase.

After the experimental manipulation in the Clap & Tap Game, the children took part in the Affiliation Measure for a second time, before commencing to play both the Imitation and Sharing Measure. The order of the Imitation and Sharing Measure was counterbalanced.

#### 2.2.3 Imitation measure

**Figure 3:**
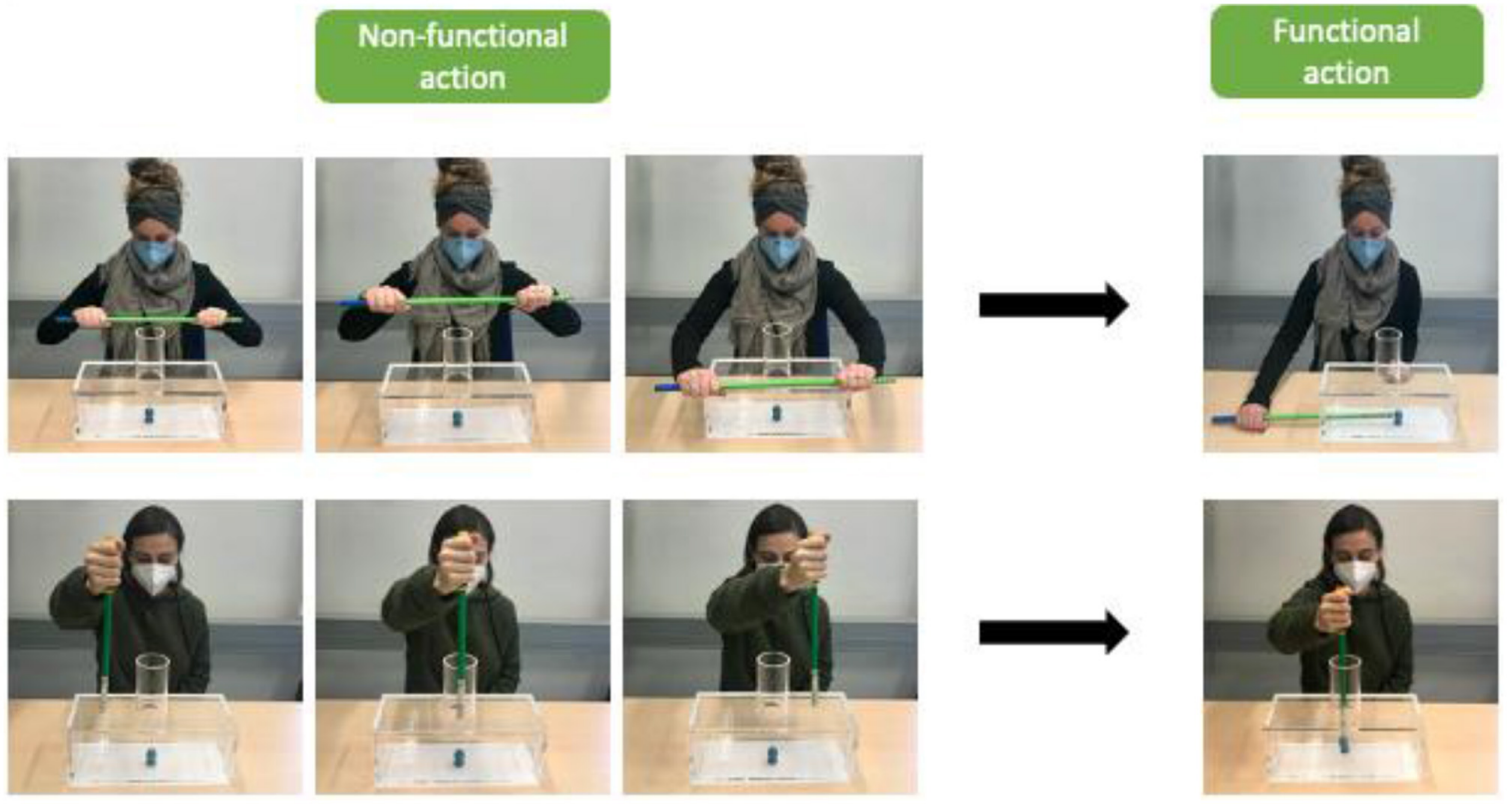
” The fish box and actions performed by the experimenters. The fish will be placed in the middle of the box. Both demonstrations consist of one non-functional action **(left)** and one functional action each **(right)**.

In the Fish Box Imitation Game, children were required to retrieve a fish from a transparent box (see Figure 3). There are two possible openings and matching sticks to retrieve the fish. The game commenced with either the SY or AS (order counterbalanced) introducing the box and the fish inside by saying: “There is a fish in this box. I am going to retrieve it using this long/short stick. Look, this is how I do it.” The experimenter went on to show one non-functional action and then one functional action (fixed order) to retrieve the fish figurine (e.g., non-functional: hold the stick horizontally with both hands and guide the stick from the back to the front over the transparent box and back, accompanied by whoop sound to highlight the action and its intentionality; functional: push the stick through a small hole on the side of the box and push the fish out through a small door on the other side), and subsequently showed the fish to the child. The first experimenter then indicated that it was the second experimenter's turn next by saying: “It's your turn now.” Next, the second experimenter reset the fish box behind a cloth and then said the same lines as the first experimenter but used the other stick, an alternative non-functional action and an alternative functional action to take out the fish (e.g., non-functional: circle stick around the cylindrical opening on top of the box, accompanied by whoop sound; functional: insert the stick through a cylinder on the top of the box and pull the fish out through the cylinder). Both non-functional actions were non-contact actions, meaning they were not in contact with the box to make it clear that they are distinct from the fish box and serve no function of reaching the overt goal of retrieving the fish (Schleihauf and Hoehl, 2020). Still, non-functional and functional actions were object-directed. After the demonstration of both the SY and AS, the experimenters each laid down the stick they used in front of the child. The first experimenter told the child that it was their turn next to retrieve the fish. The second experimenter told the child to retrieve the fish in whatever way they liked to do it. While the child retrieved the fish from the fish box, the experimenters went behind the curtain allegedly to fix something on the computer, and the child would notify them when done. The handling of the box and instructions were divided equally across both experimenters. According to our pilot study, the box was suitable for this age group as all children (*N* = 17) managed to retrieve the fish using one of the shown functional actions.

#### 2.2.4 Sharing measure

The Sticker Sharing Game assessed children's sharing behavior and represented an adapted, age-appropriate Dictator Game (Benenson et al., 2007; Steinbeis and Over, 2017). There were nine stickers on the table and three envelopes, one for each experimenter and one for the child with names prewritten on the envelopes. One experimenter started the game by putting all three envelopes on the table, and the other selected their own envelope and handed the child's envelope to them. One of the experimenters then instructed the child by counting the stickers. The two experimenters each put two stickers into the child's envelope, closed it, and laid it next to the child. Then they instructed the child to distribute the remaining five stickers into the experimenters' individual envelopes and post the envelopes in a post box. The child was told about the anonymity of their decision. The experimenters went behind the curtain until the children called them back when they were finished with the distribution.

### 2.3 Coding and reliability

*Affiliation measure*. The children's chosen proximity to SY and AS was binary (1 when children chose to sit next to SY, 0 when children chose to sit next to AS). The affiliation scores from before the clap & tap game (Affiliation at T1) were analyzed to control for experimenter preference and the analyses were conducted separately from the affiliation score after the clap & tap game (Affiliation at T2).

*Imitation measure*. Functional imitation was coded in a binary manner (1 when the SY is imitated, 0 when the AS is imitated). Children's over-imitation scores were considered independently of their imitation scores. Over-imitation of at least one of the non-functional actions was coded in a binary manner (1 when the SY was over-imitated, 0 when the AS was over-imitated or neither SY nor AS were over-imitated). If the child did not produce any of the functional actions within 30 s, we kindly nudged the child to engage with the box. All children extracted the fish within 90 s.

*Sharing measure*. The distribution of stickers was coded binary (1 when the SY receives more stickers than the AS, 0 when the SY receives fewer stickers than the AS). If the child failed to distribute the stickers preferentially, by either keeping all stickers for themself or failing to allocate the fifth sticker, the case was considered as a dropout for this dependent variable (*N* = 1).

Children's behaviours were coded based on edited video recordings showing only the child's choice of seat, the child trying to extract the fish from the box as well as the child's distribution of the stickers. An additional independent coder (ignorant to the condition and role of the experimenters) also coded 30% of the videos. Interrater reliability was assessed using unweighted Kappa and the independent coders were trained until interrater reliability over >.80 was reached. Both coders reached perfect interrater reliability for all dependent variables, = 1.00.

### 2.4 Analysis pipeline

Data were analyzed using sequential hypothesis testing using Bayes Factor^1^ [see Schönbrodt and Wagenmakers (2018) and Mani et al. (2021) for further details and interpretation of BF values]. We hypothesized that motor synchrony will increase children's social affiliation with their interaction partners. Accordingly, children were expected to be more likely (1) to affiliate with, (2) to imitate, (3) to over-imitate, and (4) to share with partners who had moved in synchrony with them as compared to partners who had moved asynchronously with the child.

To test hypotheses 1–4, we conducted a proportion analysis using the function *proportionBF* of the package BayesFactor (Morey et al., 2015) in RStudio (RStudio Team, 2020). To test our hypotheses, we continuously conducted four separate Bayesian proportion tests from when the initial sample size reached 36 participants (balanced for biological sex) and for each additionally tested participant. We continued to try to ensure the equal occurrence of biological sex of participating children (taking into account scheduling constraints).

To control for initial preferences toward either experimenter, we tested the proportions of children's preference for sitting close to SY before the synchrony manipulation against chance level. Evidence for a proportion score around chance level would indicate no preference for SY or AS. To test hypothesis 1, we then tested the proportions of children's preference for sitting close to SY after the synchrony manipulation against chance level. Evidence for a proportion score above chance level would indicate a preference for SY. For hypothesis 2, children's proportion of imitation of SY was tested against chance level. To test hypothesis 3, we tested the proportions of SY over-imitation against chance level. Hypothesis 4 tested proportions of prosocial sharing (i.e., more) toward SY against chance level. We assumed the prior scaling parameter of Cauchy r = 1/2 (medium scaling constant), based on effect sizes from previous research (Tunçgenç and Cohen, 2018; Cross et al., 2021). Data collection was planned to be stopped once the Bayes Factor (BF) for key hypothesis 2 reached 6 (moderate evidence for H1) or 0.167 (moderate evidence for H0) (Schönbrodt and Wagenmakers, 2018; Mani et al., 2021). Moreover, we planned to stop data collection at 88 participants considering the statistical power analysis (see below), plus oversampling to account for an estimated 10% dropout rate. Accordingly, we stopped data collection at *N* = 86, as BF for hypothesis 2 reached < 0.167.

### 2.5 Statistical power analysis

The R scripts of the analyses and power simulations can be found in the online repository at https://doi.org/10.17605/OSF.IO/RZVJS.

The key hypothesis of the current study is that motor synchrony increases social motivations regarding social learning, i.e., imitation of particular manner actions. We, therefore, conducted a simulation to evaluate the power of our planned analyses assuming different imitation levels of functional manner actions according to motor synchrony and asynchrony. We tested different probabilities of imitation of the two experimenters. The probability of imitation was either 70% {Odds Ratio [OR] = 2.3; small effect size (Cohen, 2000)}, 75% (OR = 3; medium effect size), or 80% (OR = 4; large effect size). We generated the response variable by randomly sampling from a binomial distribution (*rbinom*) using the three assumed probabilities of imitation of a functional manner action.

We simulated 1,000 datasets (with 20–88 subjects per dataset and one observation per subject) and Bayesian proportion tests. We extracted the Bayes Factor out of all 1000 simulated model comparisons and evaluated at which sample size the Bayes Factor reached 6 or 1/6. Overall, we found moderate evidence (BF ≥ 6) for the motor synchrony effect in 90% of the simulations at a minimum sample size of 36 (large effect size), 62 (medium effect size), and 80 (small effect size). The results were inconclusive (1/6 < BF < 6) in 0.1–7.9% of the simulations. None of the simulations resulted in a false-negative outcome (BF ≤ 1/6). Overall, the simulations revealed sufficient power (1–β = 0.9) to examine the motor synchrony effect within a sample size range of 36 to 80 in the present experiment.

## 3 Results

### 3.1 Data quality check

Firstly, we wanted to check that the data from multiple tasks were independent of each other and that individual differences unrelated to the synchrony manipulation did not account for children's responses. In these analyses, we conducted Bayesian logistic regression and additional binomial analyses of all dependent variables [affiliation, (over)imitation, and sharing behavior] and tested for order effects.

**Figure 4:**
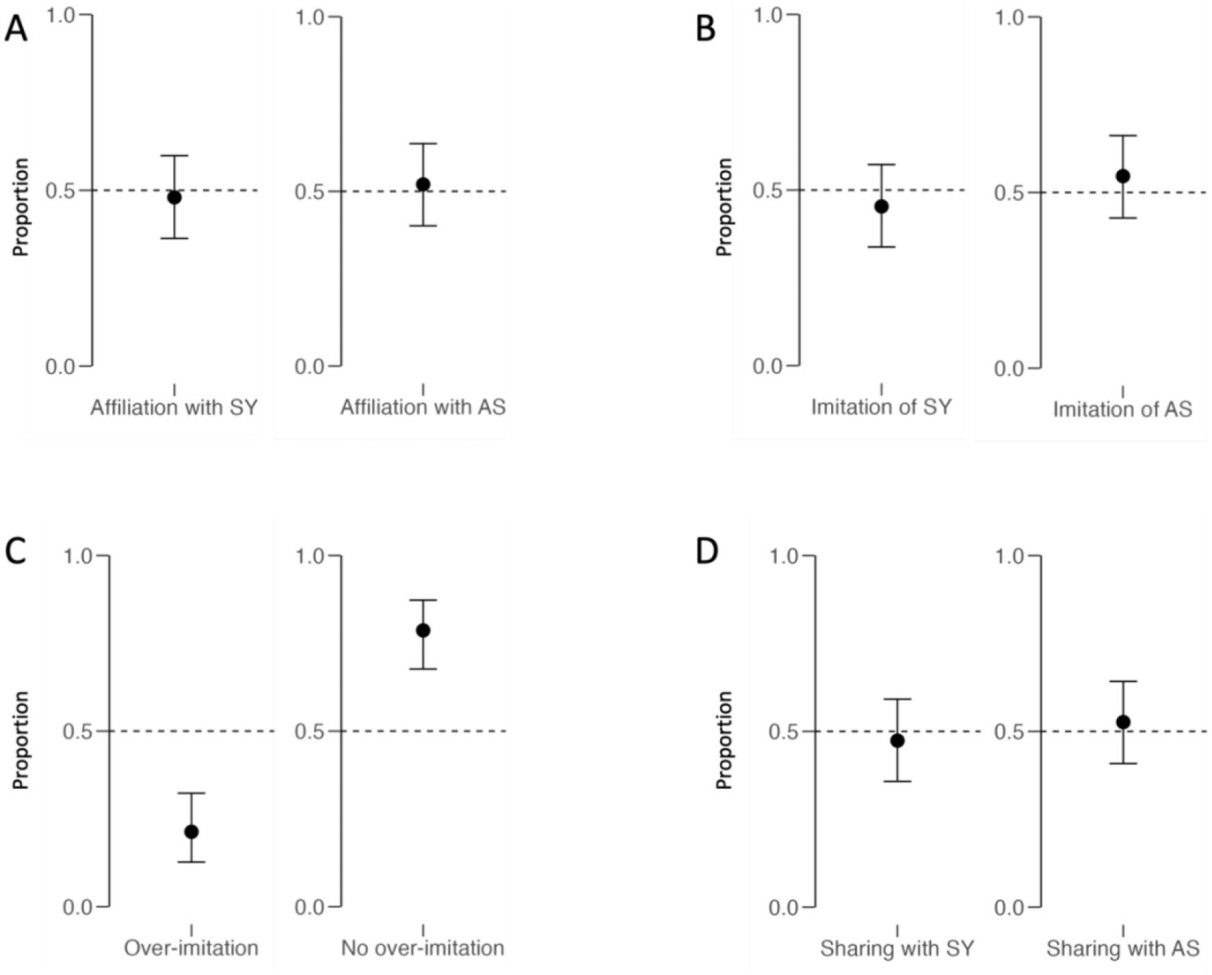
Graph depicts descriptive plots of the proportion of times that each dependent variable was observed including 95% CI. **(A)** depicts children's Affiliation at T2. **(B)** shows children's imitation behaviour. **(C)** shows children over-imitation behaviorof SY. **(D)** shows children's sharing behaviour.

*Affiliation and sharing*. We found that the initial affiliation of children toward the experimenter predicted children's sharing behavior toward the same person (*coef* = 1.119, *SD* = 0.440, BF_10_ = 14.781, Figure 4), with strong evidence for the H1. This means that if children showed preferences toward SY from the beginning, they also shared more stickers with SY and vice versa for AS. To account for children's initial preference, we thus included affiliation at T1 in the confirmatory analysis including covariates.

*Imitation and over-imitation*. Secondly, we found extreme evidence for H1 regarding the correlation between imitation and over-imitation (*coef* = 2.250, *SD* = 0.746, BF_10_ = 250.675). If children imitated SY, they were also likely to over-imitate SY's actions as well, and vice versa for AS.

There were no further significant correlations between other combinations of the dependent variables, and we did not find substantial order effects (see Supplementary S2, S3 for full details).

### 3.2 Confirmatory analyses: testing the effects of synchrony

**Figure 5:**
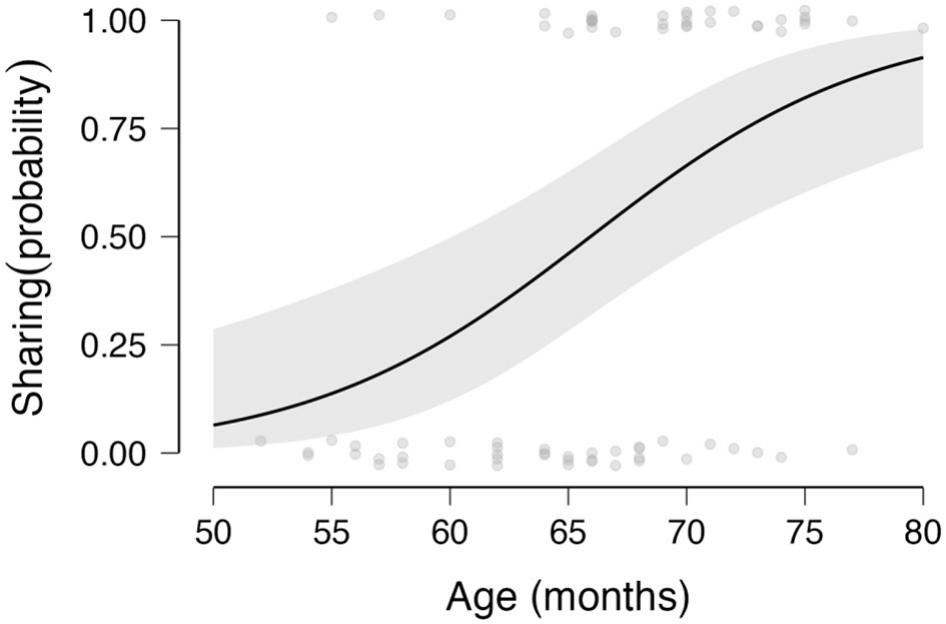
Graph depicts the main effect of age (x Axis) on children's sharing behavior(y Axis, with SY = 1, with AS = 0). The older children were more likely to share more stickers with SY than with AS.

We then ran the initially registered simple Bayesian binomial tests and results are visualized in Figure 5.

*Affiliation*. Children (*N* = 75) showed no differences in initial affiliation toward SY (*n* = 36) or AS (*n* = 39) before the experimental manipulation, which confirms that experimenters correctly adhered to the protocol and the randomization of experimenters being SY or AS was effective. However, they also did not show a difference in affiliation toward SY (*n* = 36) nor AS (*n* = 39) after the manipulation. The results provide moderate evidence for H0 in both analyses (BF_10_ = 0.216).

*Imitation*. Next, we tested whether children imitated SY more often than AS. Children imitated AS in 41 cases and only imitated SY in 34 out of 75 cases, thus providing moderate evidence for H0 (BF_10_ = 0.163).

*Over-imitation*. Children showed over-imitation of SY in 16 and over-imitation of AS in 19 out of 75 cases. Forty children did not show over-imitation at all.

The results thus provide strong evidence for the H0 (BF_10_ = 0.045).

*Sharing*. Children showed little evidence of preferential prosocial behavior toward either SY (*n* = 34) or AS (*n* = 40). The results point toward moderate evidence for H0 (BF_10_ = 0.187).

### 3.3 Exploratory analyses

Given that we did not find evidence for our hypothesised effects, we checked whether the effects were influenced by child demographics and whether the synchrony manipulation was successful.

#### 3.3.1 Testing the effects of sex, age, and initial affiliation

We conducted Bayesian logistic regressions to include age and sex as predictors for children's affiliation response at T1 and T2, imitation, over-imitation and sharing behaviour. The addition of age and sex provided moderate to strong evidence for the H0 (BF_10_ = 0.081-0.154), thus indicating that age and sex did not play a role in children's affiliation, imitation, and over-imitation responses.

**Figure 6:**
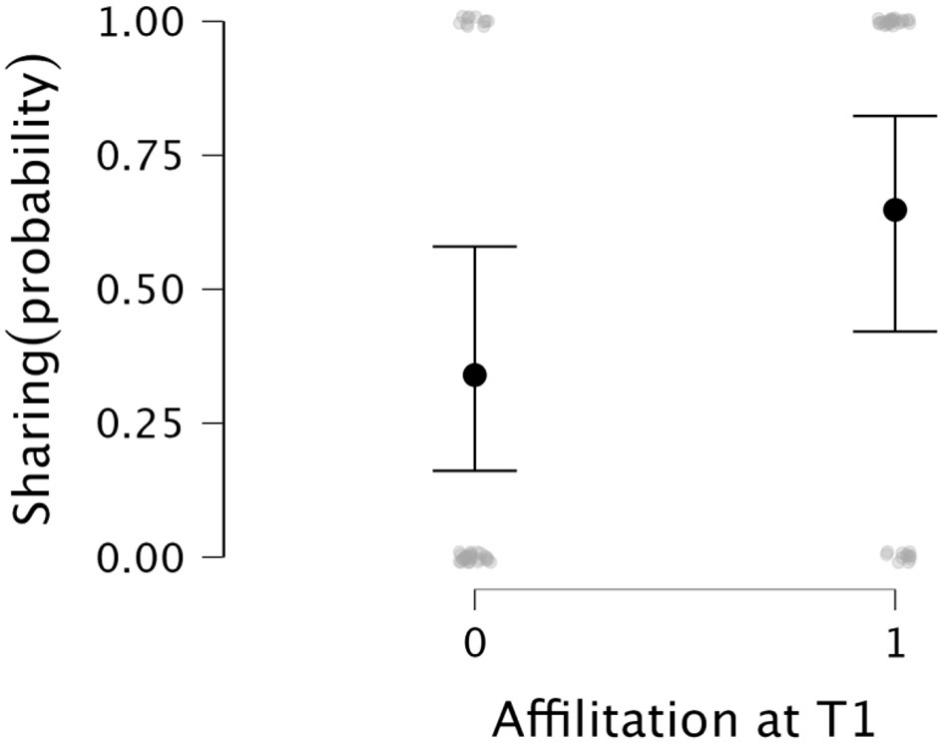
Graph depicts the main effect of Affiliation at T1 (x Axis) on children's sharing behaviorwith SY (y Axis; SY = 1, with AS = 0). The children who preferred SY at the beginning of the experiment were more likely to share more stickers with SY than with AS.

In contrast, the Bayesian logistic regression on children's sharing behavior revealed that children were more likely to share the majority of their stickers with SY than AS when they were older (*coef* = 0.149, SD = 0.052, 95% CI = 0.045–0.258, BF_10_ = 96.055, Figure 6) in addition to the previously reported effect of affiliation at T1 on children's sharing behavior.

#### 3.3.2 Motor synchrony manipulation check

We conducted *post-hoc* motion tracking analysis to examine the degree of synchrony children displayed with each experimenter and found that the synchrony and asynchrony manipulations were indistinguishable from each other in terms of coherence (coh_SY_ = 0.633, coh_AS_ = 0.621, BF_10_ = 0.380, for further details, see Supplementary). Importantly, these coherence values stand in-between the coherence values we found in our pilot data. These differences may stem from the pilot data being collected in a dyadic setting, in which the children interacted with only one adult at a time (coh_SY_ = 0.84, coh_AS_ = 0.45) and/or using different measurement devices to calculate the coherence values (acceleration trackers for pilot data vs. DeepLabCut for current study). We further extracted the tempi of the movements performed by the children, SY and AS using both DeepLabCut and verified these by manually tapping along to the videos to examine a more liberal form of coordination. The results aligned and we found that SY and AS adhered to their instructed clap-and-tap rhythm and showed clear movement tempi differentiation from each other (tempo_SY − AS_ = 25.95 BPM, SD_SY − AS_ = 4.33 BPM). The tempo difference is slightly less than 30 BPM due to the whoop motions that interrupted the clap-and-tap motions. On the other hand, children seemed to clap and tap to a tempo in-between both experimenters but were still closer to the tempo of SY than AS (tempo_CH − SY_ = 7.86 BPM, SD_CH − SY_ = 7.73 BPM; tempo_CH − AS_ = 22.46, BPM, SD_CH − AS_ = 10.09 BPM). Bayesian Paired Samples Wilcoxon Signed-Rank Tests were used to compare tempo difference values between child-SY and child-AS pairings and suggest strong to extreme evidence for robust differences between all pairings [BF_10_ = 75.95–1.182 x 10^6^]. Taken together, the lack of differentiation in the child-SY and child-AS coherence values as well as the variance in children's movement tempo suggest that overall low levels of synchrony were attained. To understand potential reasons for this, we examined whether the motor synchrony might have been more enhanced in older children or dependent on their biological sex. Motor synchrony was not higher in older children (*r* = −0.073, BF_10_ = 0.152) nor was it dependent on biological sex (BF_10_ = 0.772). Similarly, tempo differences from SY were not lower (or tempo differences from AS were not higher) in older children (BF_10_ = 0.257–0.280) nor were those differences related to biological sex (BF_10_ = 1.000). Overall, these analyses indicate that relatively low levels of synchrony were attained in the three-person group setting of the current study, and that there may not have been sufficient differentiation in how much the children synchronized with the SY vs. the ASD experimenter.

#### 3.3.3 Degree of motor synchrony as a dimensional variable

Next, we used Bayesian mixed effects modeling (using the functions *glmer* and *Bf* (Silvey et al., 2021)] to examine how the degree of motor synchrony attained predicted our dependent variables. To that end, we used the coherence values extracted from DeepLabCut as a dimensional measure of motor synchrony.

*Affiliation*. We found no credible evidence that the degree of motor synchrony attained was associated with affiliation at T1 (BF_10_ = 0.993) and anecdotal evidence for a positive association with affiliation at T2 (BF_10_ = 1.232, *estimate* = 4.063, SE = 2.199, 95% CI = [0.017 8.752]), suggesting some, albeit weak, support for H1.

*Imitation*. We found anecdotal evidence for H1 (BF_10_ = 1.349) such that there was a negative association between the degree of motor synchrony attained and imitation (*estimate* = −4.009, SE = 2.151, 95% CI = [−8.585 −0.026]).

*Over-imitation*. We found no credible evidence for an association between the degree of synchrony attained and whom the children over-imitated (BF_10_ = 1.000).

*Sharing*. We found no credible evidence for an association between the degree of motor synchrony attained and children's sharing behavior (BF_10_ = 0.999).

## 4 Discussion

In this study, we set out to test the role of motor synchrony during a clapping game for social learning, specifically focusing on imitation, over-imitation, affiliation, and sharing behavior in preschool-aged children. We designed a group setting, wherein a child engaged with two experimenters—one tasked with moving in synchrony (SY) and the other moving asynchronously (AS) with the child. However, our *post-hoc* analyses revealed that the SY and AS conditions were indistinguishable in terms of actual motor synchrony, indicating that the children did not spontaneously synchronize as intended. This lack of clear differentiation in synchrony likely impacted the outcomes of our study. Consistent with this, but contrary to our hypotheses, our analyses showed no credible evidence for differences in children's affiliation, imitation, over-imitation, or sharing behavior toward either experimenter. These findings suggest that the attentional and motor demands of the motor synchrony game in a group setting may have constrained the social affordances associated with interpersonal motor synchrony during play at this developmental stage.

Contrary to our initial expectations, the children in our study did not exhibit credible evidence of differences in social behaviors, such as affiliation, (over-) imitation, or sharing behavior, toward either experimenter following the experimental manipulation using a clapping game. This finding is particularly intriguing when considering the broader psychological questions related to the positive role of motor synchrony in fostering social bonding, enhancing social learning, and modulating prosocial behaviors in early childhood. The clapping game was designed to explore whether motor synchrony - where participants move together in time - would influence children's social learning by encouraging affiliation, imitation, or prosocial actions. However, the absence of behavioral differences suggests potential limitations in the effects of motor synchrony in a group context, where shared rhythmic activity might not readily translate into dyadic bonds or observable social behaviors. Our results suggest that the group setting may have posed challenges for the children, as movement phase coherences between the child-experimenter pairs did not show robust differences (see Supplementary Figure S1), prompting further exploration of how synchrony in different forms and contexts might influence social outcomes in young children.

In this group setting, children were required to synchronize their movements with an audio track played through their headphones, while simultaneously observing two experimenters sitting in front of them who moved either in or out of synchrony with them. This task demanded that children maintain their tempo (motor control) while also discerning which experimenter was moving in or out of sync with them and/or the audio track, creating significant attentional demands. Further, the task posed social demands, as the presence of two adults may have introduced ambiguity regarding whom to attend to and whom to follow. This scenario contrasts with prior studies that examined similar effects in simpler, dyadic interactions (Kirschner and Tomasello, 2010; Tunçgenç and Cohen, 2016; Cirelli et al., 2014a; Kirschner and Tomasello, 2008), where the manipulation of synchrony was conducted in a between-subjects design. The complexity of the group setting likely affected the children's ability to perceive and respond to the synchrony cues. Social effects of motor synchrony often rely on visual cues (Khoramshahi et al., 2016; Miyata et al., 2017; Howard et al., 2021), which may have been more challenging for children to discern in this multi-person setup. Indeed, some interactions may have appeared indistinguishable to the children, reducing the effectiveness of the synchrony manipulation. Additionally, the development of multifocal attention, which enables individuals to process multiple sources of information simultaneously, increases significantly after the preschool years (Blankenship et al., 2020) and continues to refine into adulthood (Bamford et al., 2023). In our study, the subtlety or lack of clear contrast between the synchronous and asynchronous movements may have been too difficult for preschool-aged children to detect. This challenge could have interfered with their ability to maintain the rhythm, stay in synchrony, and direct their attention toward the synchronous experimenter throughout the game (Keller et al., 2014).

This outcome highlights the importance of considering the developmental stage and the complexity of the game in group settings when investigating the psychological effects of play and games on social behaviours. While games, such as the clapping game, have the potential to foster social connections and enhance learning, the effectiveness of such activities may vary depending on the cognitive and attentional capacities of the participants, which may affect the degree of synchrony they are able to attain with their interaction partners. Indeed, we found some, albeit weak, evidence that those children who attained higher degrees of synchrony affiliated more with their interaction partner. However, the levels of synchrony were low and indistinguishable between the two pairs overall. This suggests that at preschool age and younger, only monofocal contexts of motor synchronisation may induce the positive social effects found in previous studies (Tunçgenç and Cohen, 2018; Cirelli et al., 2014a). Understanding these nuances is crucial for designing play-based interventions and activities that maximize positive social and cognitive outcomes across different age groups and settings.

Beyond clear instances of joint actions, such as frequency and phase matching of rhythmic movements typically studied in dyadic interactions (Sebanz and Knoblich, 2021), it is increasingly recognized that true social coordination is far more complex. Interpersonal synchrony often extends beyond temporal alignment to encompass contingent, bidirectional, and multimodal coordination, where partners respond flexibly and reciprocally across multiple channels of interaction. These forms of synchrony, such as cross-modal coordination, play significant role in social interactions and are observed not only during early interactions in infancy (Markova and Nguyen, 2023) but also in our broader physical environment, such as in the synchronization of visual and audio stimulation during dance (Wass et al., 2020; Bigand et al., 2024). These forms of synchrony are thought to be more socially meaningful and predictive of developmental outcomes than frequency and phase matching alone (Markova and Nguyen, 2023; Leclère et al., 2014; Wass et al., 2024). Reflecting these distinctions, our study implemented an experimental setup in which the audio track played to the children was synchronized with the movements of one experimenter but not the other, irrespective of the children's own actions. This setup may have led children to perceive one experimenter as more adept at synchronizing with the audio, thus appearing more competent in the context of the game.

It could be argued that although an insufficient degree of interpersonal motor synchrony was attained, the children could have observed the SY experimenter's movements to be in synchrony with their own audio track and responded to this cross-modal (i.e., visual – auditory) contingency. The fact that older children in our sample were more likely to share with the SY experimenter suggests that as children develop, they become more attuned to these cross-modal cues, incorporating them into their decision-making processes (Rizzo et al., 2016). In contrast, younger children may require more easily predictable rhythms to fully benefit from the social advantages of interpersonal synchrony (Hoehl et al., 2021; Nguyen et al., 2023).

Overall, these findings underscore the need for further investigation into the developmental trajectory of motor synchrony effects, particularly in the context of play. Understanding how children's sensitivity to synchrony and coordination develops in dyadic and group interactions can provide valuable insights into how different types of play activities—whether they involve direct physical coordination or more subtle forms of synchrony—contribute to social learning and development across the lifespan.

Next, we would like to highlight the lack of consistency between the children's responses in social learning tasks (imitation and over-imitation) and those in tasks measuring affiliation and sharing behavior. Our study design provided children with a forced choice between two experimenters across different domains of social cognition, using a series of playful tasks. However, in the absence of a clear social cue, such as synchronous movement to guide their choices, children's responses were not consistent across these domains (see Sections 3.1 and Supplementary S2, S3). Notably, only imitation and over-imitation were strongly correlated, suggesting that these behaviors may be assessing very similar constructs to each other that are distinct from affiliation and sharing behavior (Wilks et al., 2019; Mackie et al., 2024). These findings demonstrate that it is both possible and valuable to examine multiple social cognition measures within the same participants, without necessarily inducing carry-over effects from one task to another.

Interestingly, we found that initial affiliation and sharing behavior were related, which aligns with previous research showing that feelings of affiliation can motivate helping and comforting behaviors in children (Giner Torréns and Kärtner, 2019). This relationship suggests that children's choices in resource allocation may have been influenced by their initial preferences or first impressions of the experimenters, reflecting a tendency to stay loyal to these early perceptions (Cook et al., 2022). This result, though preliminary, suggests that even subtle initial preferences formed during play can have a lasting impact on children's prosocial behavior in subsequent games and tasks. Future studies should consider the role of initial preferences and the specific types of play activities that can foster or hinder prosocial behaviour.

In summary, our study sheds light on the multifaceted relationship between motor synchrony and social development in early childhood. Contrary to our hypotheses, we found no significant differences in children's social behaviors in the synchronous vs. asynchronous conditions, which, as described above, did not reliably induce motor synchrony. This suggests that in a group interaction setting, the attainment of motor synchrony, and any subsequent social effects it can incur may be constrained by attentional and motor demands, with prosocial effects emerging as children develop. Further research is needed to explore the developmental trajectory of motor synchrony effects in the context of play and games, considering age-related differences in social learning and prosocial behaviors. This study opens avenues for future investigations into the nuanced interplay between motor synchrony and social development during early childhood.

## Data Availability

The datasets presented in this study can be found in online repositories. The names of the repository/repositories and accession number(s) can be found at: https://doi.org/10.17605/OSF.IO/RZVJS.

## Footnotes

^1^Using a frequentist approach, multiple testing of the same data leads to Type I error inflation (Armitage et al., 1969). We can avoid some of the pitfalls of the frequentist approach when using Bayes Factor analyses. More specifically, the inference criteria in frequentist and Bayesian statistics develop differently over time due to increasing sample size. While in frequentist analysis, the *p*-value only converges to 0 when the alternative hypothesis is true. In case the null hypothesis is true, the *p*-value does not follow a systematic pattern and takes a random value between 0 and 1. In a number of tests, some of them would thus still lead to false significant results. In Bayesian analyses, if the alternative hypothesis is true, the Bayes Factor converges to infinity. Meanwhile, if the null hypothesis is true, the Bayes Factor converges to zero (i.e., consistency; Morey and Rouder, 2011; Rouder et al, 2012).
