# Supplementary material for: Motor synchrony, social learning and closeness in group play settings
Source: Front Psychol. 2025 Sep 4;16:1595908. doi: 10.3389/fpsyg.2025.1595908 (PMC12447275; doi:10.3389/fpsyg.2025.1595908)
Supplement: Supplementary file 1 [file Table_1.docx]

**Supplements for “Motor Synchrony, Social Learning and Closeness in Group Play Settings”**

**S1. Motor synchrony manipulation check**

We extracted children’s and experimenters’ movement time series by using Deeplabcut (version 2.2.3) ^1,2^. We labelled three body parts per individual (head, left, and right hand per individual) in 5 frames taken from all the videos (n=80; 95%, as per default settings, were used for training). We used a ResNet-50 neural network with default parameters for multi-subject detection for 200000 training iterations. The test error was 13.46 pixels (image size was 1920 by 1080 px). This network was used to analyse videos from the same or similar experimental settings. The coordinates were then preprocessed by demeaning the values, converting x and y coordinates to acceleration values (by differentiating twice) before excluding outliers above or below 3 SD, and using cubic interpolation to fill in missing values. We then calculated the phase coherence in the frequency band of children’s audio track for N=68 pairs of children and experimenters (Child-SY, Child-AS). Eight pairs had to be excluded as camera angles did not allow for accurate tracking, as either experimenters or children were obstructed from view.

Using a Bayesian paired t-test, we calculated whether child-SY pairs showed higher motor synchrony than child-AS pairs. The results indicate anecdotal evidence for H0 as child-SY pairs only showed coherences slightly above child-AS pairs (see *Figure S1, Descriptives in Table S1*, BF_10_=0.380). A closer inspection of the synchrony values revealed that 42 pairs showed higher synchrony values in child-SY pairs than in child-AS pairs. Instead, 26 pairs showed higher synchrony values in child-AS pairs than in child-SY pairs and did thus not pass the manipulation check. These results underpin that the triadic motor synchrony task might have been very challenging for children. Therefore, we ran additional exploratory analyses, including coherence values.

In the first analyses, we reran all binomial tests, only including the dyads that passed the manipulation check (N=42). Still, we only find anecdotal evidence for H0, and thus, motor synchrony between children and SY still did not seem to affect children’s affiliation, (over-) imitation or sharing behaviour towards SY (BF_10_=0.061-0.580).

Secondly, we used the difference score of motor synchrony between child-SY minus motor synchrony child-AS to take into consideration whether children could have differentiated between the two experimenters irrespective of their own synchronisation to the other experimenters. In these analyses, we found anecdotal evidence for H0 as motor synchrony differences were not related to children’s affiliation, imitation, over-imitation or sharing behaviour towards SY (BF_10_=0.333-0.619).

**S2. Relation between dependent variables**

Affiliation at T1 did not predict affiliation at T2 (*coef*=0.221, *SD*=0.360, BF_10_=0.403). More specifically, out of the 36 children who stayed closer to SY in the first affiliation game, 18 remained with SY and 18 switched to AS. Out of the 39 children who stayed closer to AS, 21 remained with AS and 18 switched to SY. When we looked into whether children changed their affiliation status, we found moderate evidence for H_0_, namely that children changed or remained with their previous affiliation at around chance level (34 out of 75 children changed their affiliation after the clap-and-tap game). Out of those children only 17 children changed their affiliation towards the synchronous experimenter, thus providing very strong evidence for the H_0_ (BF_10_=0.023).

Next, we found that affiliation pre and post synchrony manipulation were not related to imitation (for both affiliation pre and post: *coef*=0.061, *SD*=0.313, BF_10_=0.325; anecdotal evidence for H_0_) or over-imitation (*coef*=-0.103, *SD*=0.383, BF_10_=0.328; anecdotal evidence for H_0_). In further analyses, we found anecdotal evidence for the H_0_ regarding a link between imitation and sharing (*coef*=0.211, *SD*=0.325, BF_10_=0.410) or overimitation and sharing (*coef*=0.120, *SD*=0.376, BF_10_=0.319). We further tested whether children who would imitate one experimenter would share more stickers with the other experimenter or vice versa. Here, we found anecdotal evidence for H_0,_ that children would imitate and share more with the same experimenter or change at chance level (*coef*=0.085, *SD*=0.232, BF_10_=0.414). These sets of results highlight that other than Affiliation at T1, dependent variables were independent of one another, thus having no further implications for the confirmatory analyses.

**S3. Testing for order effects**

Next, we conducted analyses to check for order effects. We found anecdotal evidence for H_1_ that children were more likely to imitate SY when the Fish Box Game was played after the Sticker Game (*coef*=-0.767, *SD*=0.414, BF_10_=1.722). In other words, children were more likely to imitate AS when the imitation task came right after the motor synchrony manipulation. In addition, we found anecdotal evidence for H_0_ as children’s sharing behaviour did not seem to depend on the order of the games (*coef*=0.068, *SD*=0.354, BF_10_=0.340).

**Supplementary tables**

| **Table S1. Descriptives of Coherence values (Motor synchrony)** | | | | | | | | | |
| --- | --- | --- | --- | --- | --- | --- | --- | --- | --- |
|  | | | | | | | | **95% Credible Interval** | |
|  | | **N** | | **Mean** | **SD** | **SE** | **Coefficient of variation** | **Lower** | **Upper** |
| Coherence Child-SY |  | 67 |  | 0.298 | 0.127 | 0.015 | 0.424 | 0.267 | 0.329 |
| Coherence Child-AS |  | 67 |  | 0.282 | 0.127 | 0.016 | 0.450 | 0.251 | 0.313 |
|  | | | | | | | | | |

**Supplementary Figures**

**Figure S1. Raincloud plot of Coherence values**

**
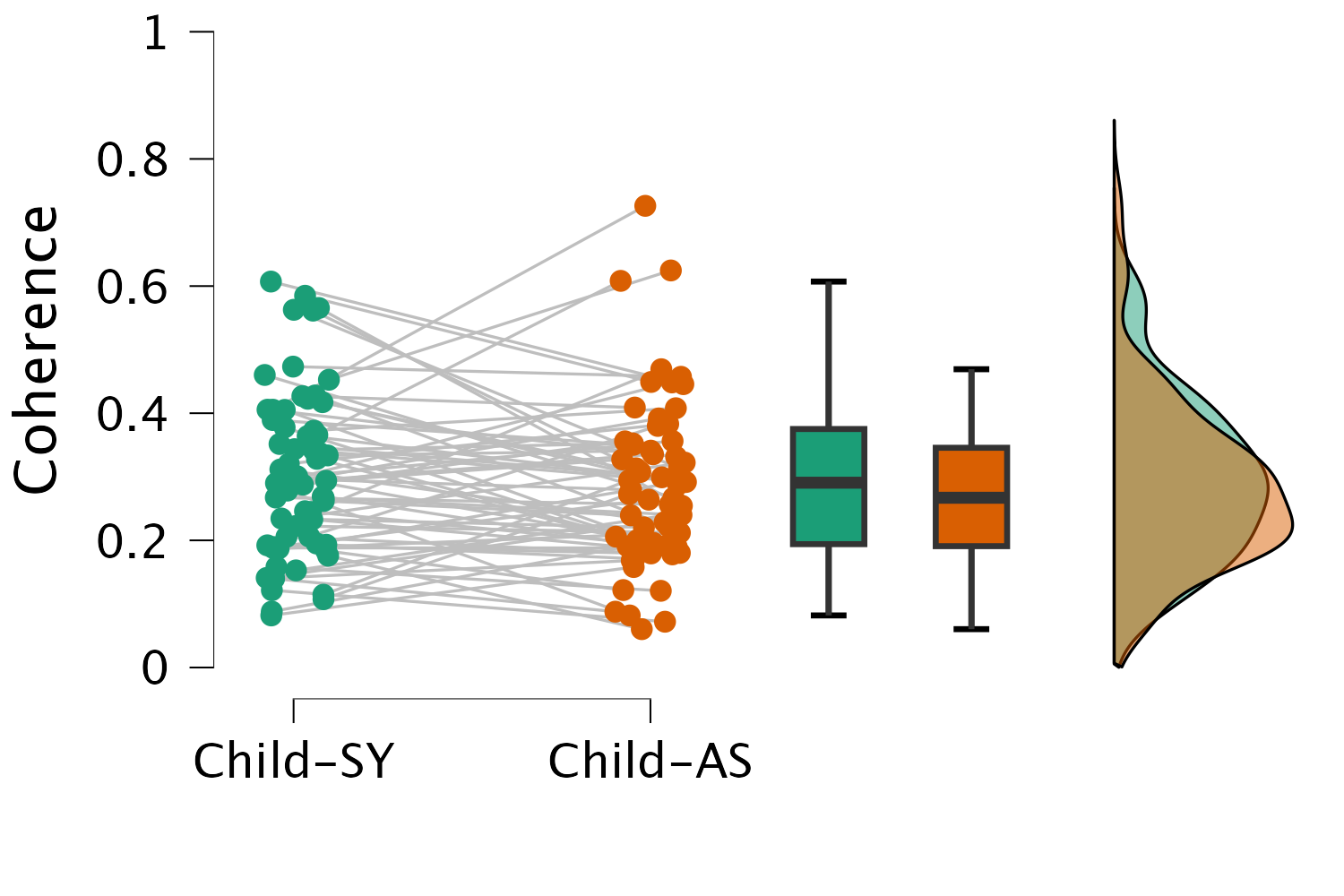
**

**References**

1. Mathis, A. *et al.* DeepLabCut: markerless pose estimation of user-defined body parts with deep learning. *Nat. Neurosci.* **21**, 1281–1289 (2018).

2. Nath, T. *et al.* Using DeepLabCut for 3D markerless pose estimation across species and behaviors. *Nat. Protoc.* **14**, 2152–2176 (2019).
